# Predicting Anxiety and Depression Among Patients With COVID-19 in Concentrated Isolation at Medical Camps in Vietnam: A Descriptive Cross-Sectional Study

**DOI:** 10.3389/fpsyt.2022.823586

**Published:** 2022-05-31

**Authors:** Vu Thi Thu Trang, Khoa Le Anh Huynh, Huyen Thi Truong, Hue Thi Nguyen, Giang Truong Hoang, Dat Quang Dao, Ut Van Vu, Zair Hassan, My Ngoc Ha Nguyen, Le Van Truong

**Affiliations:** ^1^Acupuncture Department, National Hospital of Traditional Medicine, Hanoi, Vietnam; ^2^Department of Biostatistics, School of Medicine, Virginia Commonwealth University, Richmond, VA, United States; ^3^Traditional Medicine Hospital, Ministry of the Public Security, Hanoi, Vietnam; ^4^U Buou Hospital, Bac Giang City, Vietnam; ^5^Son Uyen General Hospital, Bac Giang City, Vietnam; ^6^Lady Reading Hospital, Peshawar, Pakistan

**Keywords:** depression, COVID-19, anxiety, multiple logistics regression, medical camps

## Abstract

**Introduction:**

This study aims to assess the requirement for anxiety and depression treatment for patients with coronavirus disease 2019 (COVID-19) in medical camps in Bac Giang province, Vietnam. This information can help improve the government policy to reduce anxiety and depression in patients with COVID-19.

**Methods:**

A total of patients with 427 COVID-19 participated in the survey conducted from 5 to 15 June 2021 in Bac Giang province. The survey included 17 questions about the general characteristics of the patients, 15 questions to assess common COVID-19 symptoms, the Patient Health Questionnaire-9 (PHQ-9), and General Anxiety Disorder-7 (GAD-7) scores, and four questions to assess hospital reviews, including facilities, food, medical staff, and living conditions. Logistics regression analyses were conducted to assess the association between COVID-19 symptoms and high anxiety and depression (HAD) status.

**Results:**

A logistic regression analysis evaluated the risk factors in need of intervention. Our study showed that lower hospital review scores (odd ratio = 0.98; 95% confident interval = 0.97–0.99) were found to be a risk needing intervention. It was also identified that older patients (odd ratio = 1.1; 95% confident interval = 1.03–1.18), women (odd ratio = 1.31; 95% confident interval = 1.09–1.31), patients who were primary income earners in the family (odd ratio = 1.15; 95% confident interval = 1.03–1.28), patients who had headaches (odd ratio = 1.16; 95% confident interval = 1.06–1.21), and patients who had joint pain (odd ratio = 1.17; 95% confident interval = 1.06– 1.3) were risk factors for HAD status.

**Conclusion:**

Our research shows that every 10-year age increase was associated with a 10% increase in the likelihood of HAD status. Study subjects being primary income earners were also associated with a 15% increased risk of having HAD status. This study showed that a decrease in family income due to COVID-19 caused an increase in high-level anxiety/depression status.

## Introduction

On 1 December 2019, the first patient infected with severe acute respiratory syndrome-coronavirus 2 (SARS-CoV-2) was reported in Wuhan, China, and was described with strange pneumonia (1, 2). While complex transmission mechanisms had often been mentioned for SARS-CoV-2, such as surface contact and fecal-oral or airborne transmission (3, 4), the main transmission would in fact be direct human-to-human (3, 4). Four months after the first infected patients, SARS-CoV-2 had spread globally and became a humanitarian disaster (5, 6).

Although the first COVID-19 death occurred on 22 January, 2020, in Wuhan, China, the Vietnamese government immediately took strong measures to control the situation. Early decisions included social distancing and the isolation of all infected and suspected cases. This allowed Vietnam to control the average daily number of infections to about 6.2 cases. Centralized isolation measures significantly reduced the spread of infection sources. Bac Giang province was under such control for more than 4 months. The fourth wave of COVID-19 at the end of April 2021, consisting of the Delta variant of the virus, caused an outbreak in Bac Giang province. According to a report, authorities were required to isolate 5,779 F0 patients who were inflected by COVID-19, and 43,469 F1 cases who were in close contact with infected patients for 14 days (7). With merely nine hospitals in the province, only patients with severe symptoms were admitted. To meet the need for isolation, 326 medical camps were established (7), making use of sports halls, schools, factories, and so forth. However, these camps had inadequate facilities and limited living conditions. In addition, evidence showed that isolation were associated with psychological effects (8, 9). Our study was designed to assess the status of anxiety and depression in the medical camps in Bac Giang. Such an assessment could provide information regarding factors influencing the situation, which, in turn, could improve government policy to reduce depression and anxiety in patients with COVID-19.

## Materials and Methods

### Study Design and Participants

This study with a cross-sectional descriptive study was conducted in medical camps in Bac Giang province from 5 to 15 June 2021. The study excluded participants in Bac Giang Province Hospital, patients under 18 years old, and patients who refuse to download the ZALO app. We randomly selected isolated patients and conducted an online survey through Google Forms. After that, we communicated with the patients through the ZALO app to track their symptoms.

### Questionnaire Design

The survey included 17 questions about the general characteristics of the patients, 15 questions to assess common symptoms, the Patient Health Questionnaire-9 (PHQ-9), and General Anxiety Disorder-7 (GAD-7) scores. Categories of depression symptoms were defined as low depression (PHQ-9 score 0–9) and high depression (PHQ-9 score ≥ 10) (10). Categories of anxiety symptoms were defined as low anxiety (GAD-7 score 0–9) and high anxiety (GAD-7 score ≥ 10) (11). The low anxiety and depression (LAD) group included patients with low anxiety or low depression. In contrast, the high anxiety and depression (HAD) group included patients with moderate and severe anxiety or moderate and severe depression (moderate or severe). Finally, the survey participants graded the medical camps based on four criteria: facilities, food, medical staff, and living conditions on a 5-level Likert scale (1: very poor; 5: very good).

### Statistics Analysis

The Shapiro-Wilk test was conducted to test the normal distribution hypothesis for continuous variables. Comparisons were made using a *t*-test for continuous variables with normal distribution, and the Mann-Whitney U test for continuous variables with non-normal distribution. Data were presented as the number of patients (percent) for categorical variables. The Chi-square test (if the expected value in cell > 5) and the Fisher exact test (if the expected value in cell < 5) were conducted for categorical variables to compare baseline characteristics between the study groups (intervention and non-intervention). The correlation between Patient Health Questionnaire-9 and General Anxiety Disorder-7 was determined. Univariate and multivariate logistics regressions were conducted to evaluate the odds ratio (OR) and 95% confidence interval (95% CI) of factors between the intervention group and the non-intervention group. All hypotheses were tested as two tails, with a *p*-value < 0.05 considered statistically significant. Statistical analyses were performed using RStudio version 3.6.2.

### Ethical Consideration

The ethics committee approved all procedures of Traditional Medicine Hospital-Ministry of Public Security, Hanoi, Vietnam (No.664/QD-YHCT). Consent forms were attached to the survey questionnaire, and the patients only took the survey after accepting the terms of the study.

## Results

**FIGURE 1:**
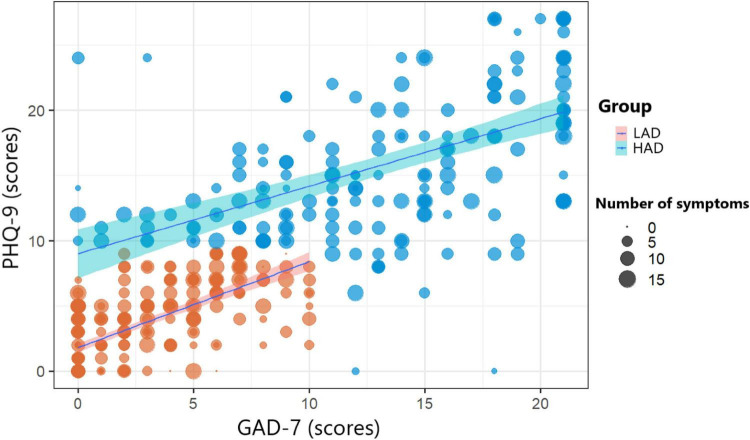
Correlation between General Anxiety Disorder-7 (GAD) and Patient Health Questionnaire-9 (PHQ) scores according to anxiety and depression status and number of symptoms.

A total of 427 patients with COVID-19 participated in this study, with a response rate of 64.2%. Men made up 31.6% of the participants, while women made up 68.4%. The mean participant age was 27.7 (standard deviation = 7.16). Moreover, 174 patients (40.7%) were identified as HAD. Figure 1 shows a positive correlation between PHQ-9 and GAD-7 scores. Specifically, the proportion of patients with a GAD-7 score greater than or equal to 10 and a PHQ-9 score less than 10 is relatively low.

According to Table 1, the mean age of the subjects in the HAD group was 30 (6.87), which was statistically significantly higher than in the LAD group, whose mean age was 26.2 (6.95). In the HAD group, 82.8% of the patients were women, compared to the 58.5% in the LAD group. Furthermore, in the HAD group, 85.1% of patients were defined as primary workers in the family, which was significantly higher than in the LAD group (68.4%). According to the personality changes criterion, the HAD group had a significantly higher rate of irritability; 35.6% compared to the 9.49% of the LAD group. The LAD group evaluated facilities, food, living conditions, and medical staff higher than the HAD group. In particular, statistical significance was found between the two groups in facilities (*p*-value = 0.016) and food (*p*-value = 0.005).

**TABLE 1:** Sociodemographic characteristics by anxiety and depression status.

	Low anxiety and depression	High anxiety or depression	Total	*P*-value
	*N* = 253	*N* = 174	*N* = 427	
Age (years)	26.2 (6.95)	30.0 (6.87)	27.7 (7.16)	<0.001
Isolation time until survey (days)	19.8 (7.63)	20.9 (7.77)	20.3 (7.70)	0.144
Confirmed time until survey (days)	12.1 (4.59)	13.0 (5.68)	12.5 (5.07)	0.074
**Gender**				<0.001
Male	105 (41.5%)	30 (17.2%)	135 (31.6%)	
Female	148 (58.5%)	144 (82.8%)	292 (68.4%)	
**Jobs**				0.164
Manual labor	217 (85.8%)	161 (92.5%)	378 (88.5%)	
Office staff	4 (1.58%)	2 (1.15%)	6 (1.41%)	
Student	12 (4.74%)	2 (1.15%)	14 (3.28%)	
Farmer	6 (2.37%)	4 (2.30%)	10 (2.34%)	
Others	14 (5.53%)	5 (2.87%)	19 (4.45%)	
**Marital status**				0.001
Single	106 (41.9%)	43 (24.7%)	149 (34.9%)	
Married/Domestic partnership	139 (54.9%)	126 (72.4%)	265 (62.1%)	
Divorced/Widowed/Separated	8 (3.16%)	5 (2.87%)	13 (3.04%)	
**Number of relatives with COVID-19**				0.002
None	173 (68.4%)	133 (76.4%)	306 (71.7%)	
1 person	33 (13.0%)	17 (9.77%)	50 (11.7%)	
2 persons	17 (6.72%)	19 (10.9%)	36 (8.43%)	
3 persons	9 (3.56%)	4 (2.30%)	13 (3.04%)	
≥4 persons	21 (8.30%)	1 (0.57%)	22 (5.15%)	
**Level of education**				0.024
University/Master/PhD/Doctoral	6 (2.37%)	2 (1.15%)	8 (1.87%)	
College	9 (3.56%)	4 (2.30%)	13 (3.04%)	
Vocational training	7 (2.77%)	2 (1.15%)	9 (2.11%)	
High school	129 (51.0%)	78 (44.8%)	207 (48.5%)	
Secondary school	91 (36.0%)	66 (37.9%)	157 (36.8%)	
Primary school	9 (3.56%)	21 (12.1%)	30 (7.03%)	
No formal education	2 (0.79%)	1 (0.57%)	3 (0.70%)	
**Main income earner in the family**				<0.001
No	89 (35.2%)	26 (14.9%)	115 (26.9%)	
Yes	164 (64.8%)	148 (85.1%)	312 (73.1%)	
**Average income (USD)**				0.146
<130	15 (5.93%)	8 (4.60%)	23 (5.39%)	
130–220	71 (28.1%)	69 (39.7%)	140 (32.8%)	
220–440	161 (63.6%)	93 (53.4%)	254 (59.5%)	
440–660	3 (1.19%)	3 (1.72%)	6 (1.41%)	
660–880	2 (0.79%)	1 (0.57%)	3 (0.70%)	
>880	1 (0.40%)	0 (0.00%)	1 (0.23%)	
**Isolation status**				0.067
Voluntary	253 (100%)	171 (98.3%)	424 (99.3%)	
Forced	0 (0.00%)	3 (1.72%)	3 (0.70%)	
**The enneagram personality**				0.924
The reformer	3 (1.19%)	1 (0.57%)	4 (0.94%)	
The individualist	4 (1.58%)	2 (1.15%)	6 (1.41%)	
The investigator	2 (0.79%)	1 (0.57%)	3 (0.70%)	
The challenger	42 (16.6%)	22 (12.6%)	64 (15.0%)	
The enthusiast	19 (7.51%)	18 (10.3%)	37 (8.67%)	
The peacemaker	119 (47.0%)	82 (47.1%)	201 (47.1%)	
The achiever	1 (0.40%)	1 (0.57%)	2 (0.47%)	
The helper	57 (22.5%)	44 (25.3%)	101 (23.7%)	
The loyalist	6 (2.37%)	3 (1.72%)	9 (2.11%)	
**Personality changes**				<0.001
Irritability	24 (9.49%)	62 (35.6%)	86 (20.1%)	
No change	211 (83.4%)	110 (63.2%)	321 (75.2%)	
More peaceful	18 (7.11%)	2 (1.15%)	20 (4.68%)	
Review of facilities	4.12 (0.80)	3.91 (0.89)	4.04 (0.85)	0.016
Review of living conditions	3.96 (0.85)	3.82 (0.92)	3.90 (0.88)	0.125
Review of food	4.29 (0.76)	4.06 (0.89)	4.20 (0.82)	0.005
Review of medical staff	4.44 (0.72)	4.29 (0.82)	4.38 (0.77)	0.059
Medical camp review (points)	16.8 (2.75)	16.1 (3.15)	16.5 (2.94)	0.015
**Worried**				0.072
Family is stigmatized	7 (2.77%)	2 (1.15%)	9 (2.11%)	
No one to take care of children	4 (1.58%)	7 (4.02%)	11 (2.58%)	
Family finances are not secure	30 (11.9%)	27 (15.5%)	57 (13.3%)	
Personal health	156 (61.7%)	115 (66.1%)	271 (63.5%)	
Family health	38 (15.0%)	18 (10.3%)	56 (13.1%)	
Others	18 (7.11%)	5 (2.87%)	23 (5.39%)	

The HAD group had a significantly higher rate of symptoms than the LAD group, except for cough symptoms. According to Table 2, asymptomatic patients in the LAD group were 9.09%, approximately 16 times higher than in the HAD group. However, the difference between the two groups was not statistically significant in terms of cough symptoms (*p* = 0.131).

**TABLE 2:** Symptoms of coronavirus disease 2019 (COVID-19) by anxiety and depression status.

	Low anxiety and depression	High anxiety or depression	Total	*P*-value
	*N* = 253	*N* = 174	*N* = 427	
Number of symptoms	5.21 (3.44)	7.95 (3.34)	6.33 (3.65)	<0.001
Body temperature	37.4 (0.86)	37.7 (0.93)	38.4 (15.9)	<0.001
**Asymptomatic**				<0.001
Yes	23 (9.09%)	1 (0.57%)	24 (5.62%)	
No	230 (90.9%)	173 (99.4%)	403 (94.4%)	
**Fever symptom**				<0.001
No	141 (55.7%)	56 (32.2%)	197 (46.1%)	
Yes	112 (44.3%)	118 (67.8%)	230 (53.9%)	
**Cough**				0.131
No	73 (28.9%)	38 (21.8%)	111 (26.0%)	
Yes	180 (71.1%)	136 (78.2%)	316 (74.0%)	
**Shortness of breath**				<0.001
No	188 (74.3%)	81 (46.6%)	269 (63.0%)	
Yes	65 (25.7%)	93 (53.4%)	158 (37.0%)	
**Sore throat**				<0.001
No	142 (56.1%)	64 (36.8%)	206 (48.2%)	
Yes	111 (43.9%)	110 (63.2%)	221 (51.8%)	
**Headache**				<0.001
No	118 (46.6%)	31 (17.8%)	149 (34.9%)	
Yes	135 (53.4%)	143 (82.2%)	278 (65.1%)	
**Muscle pain**				<0.001
No	135 (53.4%)	43 (24.7%)	178 (41.7%)	
Yes	118 (46.6%)	131 (75.3%)	249 (58.3%)	
**Chest tightness**				<0.001
No	168 (66.4%)	76 (43.7%)	244 (57.1%)	
Yes	85 (33.6%)	98 (56.3%)	183 (42.9%)	
**Joint pain**				<0.001
No	216 (85.4%)	112 (64.4%)	328 (76.8%)	
Yes	37 (14.6%)	62 (35.6%)	99 (23.2%)	
**Diarrhea**				0.001
No	170 (67.2%)	88 (50.6%)	258 (60.4%)	
Yes	83 (32.8%)	86 (49.4%)	169 (39.6%)	
**Loss of taste**				<0.001
No	145 (57.3%)	66 (37.9%)	211 (49.4%)	
Yes	108 (42.7%)	108 (62.1%)	216 (50.6%)	
**Loss of smell**				0.028
No	81 (32.0%)	38 (21.8%)	119 (27.9%)	
Yes	172 (68.0%)	136 (78.2%)	308 (72.1%)	
**Red eyes**				0.002
No	229 (90.5%)	138 (79.3%)	367 (85.9%)	
Yes	24 (9.49%)	36 (20.7%)	60 (14.1%)	
**Loss of ability to speak**				0.002
No	246 (97.2%)	156 (89.7%)	402 (94.1%)	
Yes	7 (2.77%)	18 (10.3%)	25 (5.85%)	
**Rash**				0.016
No	220 (87.0%)	135 (77.6%)	355 (83.1%)	
Yes	33 (13.0%)	39 (22.4%)	72 (16.9%)	
**Nausea/vomiting**				<0.001
No	205 (81.0%)	104 (59.8%)	309 (72.4%)	
Yes	48 (19.0%)	70 (40.2%)	118 (27.6%)	

As shown in Table 3, HAD status was significantly higher among older patients, women, main income earners, and those suffering from shortness of breath, headaches, joint pain, and loss of speaking ability. Moreover, patients with loss of speaking ability have higher odd ratios of needing interventions than patients without this loss (OR = 1.39; 95% CI = 1.15–1.70). Besides these factors, hospitals with higher review scores had fewer patients in the HAD group (OR = 0.98; 95% CI = 0.96–1). According to the results of multiple binary logistic regression analysis evaluating the risk factors in need of intervention, lower hospital review scores (OR = 0.98; 95% CI = 0.97–0.99) were found to be a risk for needing intervention, while older patients (OR = 1.1; 95% CI = 1.03–1.18), women (OR = 1.31; 95% CI = 1.09–1.31), patients who were main income earners (OR = 1.15; 95% CI = 1.03–1.28), patients who had headaches (OR = 1.16; 95% CI = 1.06–1.21), and patients who had joint pain (OR = 1.17; 95% CI = 1.06–1.3) were identified as risk factors for HAD status.

**TABLE 3:** Multivariable regression related to indications for high anxiety and depression (HAD) status.

Variables	Univariate analysis		Multivariable analysis	
	OR (95% CI)	*P*-value	OR (95% CI)	*P*-value
Age (10 years)	1.20 (1.12–1.28)	<0.001	1.10 (1.03–1.18)	0.006
Confirmed time until survey (weeks)	1.06 (1.00–1.13)	0.063	1.04 (0.99–1.11)	0.137
Hospital review (points)	0.98 (0.96–1.00)	0.013	0.98 (0.97–0.99)	0.007
**Gender**				
Male	Reference		Reference	
Female	1.31 (1.19–1.44)	<0.001	1.20 (1.09–1.31)	<0.001
**Main income earner in the family**				
No	Reference		Reference	
Yes	1.28 (1.16–1.42)	<0.001	1.15 (1.03–1.28)	0.010
**Shortness of breath**				
No	Reference		Reference	
Yes	1.33 (1.21–1.46)	<0.001	1.10 (1.00–1.21)	0.060
**Headache**				
No	Reference		Reference	
Yes	1.36 (1.24–1.49)	<0.001	1.16 (1.06–1.28)	0.002
**Joint pain**				
No	Reference		Reference	
Yes	1.33 (1.19–1.48)	<0.001	1.17 (1.06–1.30)	0.003
**Loss of ability to speak**				
No	Reference		Reference	
Yes	1.39 (1.15–1.70)	0.001	1.19 (0.99–1.42)	0.061

## Discussion

In the face of complicated developments of the COVID-19 epidemic in Vietnam, the government has taken an uncompromising approach to the centralized isolation of all confirmed cases. The measure has shown apparent effectiveness in controlling the COVID-19 outbreak. However, our study showed that the isolation process significantly impacted mental health. Symptoms of COVID-19 were positively correlated with depression and anxiety. Our study showed that age, confirmed cases until the survey, gender, being primary income earners, shortness of breath, headaches, joint pain, and loss of speaking ability were significantly associated with the likelihood of having HAD status. Furthermore, the medical camp review was significantly associated with reduction in LAD status. Our study points to the necessity of depression and anxiety treatment, such as psychological consultations, as well as the establishment of a policy to financially support low-income patients with COVID-19.

A previous study from Kong et al. (12) showed that the PHQ-9 and GAD-7 scales increase with age for COVID-19 patients. Our findings indicate that every 10-year increase in age was associated with a 10% increase in the likelihood of HAD status. The 1-week increase after the confirmation of COVID-19 was associated with a 4% increased likelihood of having HAD status. These results were similar to Yanyu Hu’s study, which showed that the duration of COVID-19 was associated with a statistically significant increase in GAD-7 and PHQ-9 scores (13). Our results also showed that being female was associated with a 20% increased likelihood of having HAD status. This is similar to a previous study showing that women with COVID-19 have higher depression and anxiety scores than men (14–17).

In the multivariate logistics regression results, the common symptoms of shortness of breath, headaches, and joint pain were significant factors related to the HAD group. Furthermore, study subjects being primary income earners were also associated with a 15% increased risk of having HAD status. Healthcare workers also have an increased chance of exposure to COVID-19 (18). This study showed that a decrease in family income due to COVID-19 led to an increase in high-level anxiety/depression status (19). In other studies, asymptomatic patients at the time of testing accounted for 30.8–51.7% (20, 21); this rate differed from our study (5.62%). However, follow-up studies over time show that the proportion of asymptomatic patients was much lower at about 10.7 (22) to 15.6% (23). This difference could be explained by patients being diagnosed at an early stage when they were not showing any symptoms.

Furthermore, a 1-point increase in the medical camp review was associated with a 2% reduction in the likelihood of having HAD status. During the isolation period, patients with COVID-19 shared a small space with limited facilities with other patients with COVID-19. Therefore, it was not surprising that their satisfaction with the medical camp was significantly associated with reduction in HAD status.

There were some limitations to our study. First, although this study was a random selection of patients through an online format, most of the participants were relatively young (mean age was 27.7); thus, it is necessary to investigate with an older participant pool. Second, there is a lack of follow-up in the study design related to depression and anxiety. Follow-up studies would clarify participants’ levels of depression and anxiety after being COVID-19-free. Third, this study was conducted based on a self-reported questionnaire, which may involve respondents’ bias.

## Conclusion

This study indicated that increase in age would increase the likelihood of HAD. The advantage of medical camp has reduced the severity of shortage number of beds in the hospital, low-cost facilities and easy to establish. Moreover, low income and being a primary income earner also increased the risk of having HAD status. Government and policymakers should, thus, offer financial support for low-income patients with COVID-19. Our study further showed that HAD status was correlated with increase in symptoms of COVID-19. Therefore, psychological consultations in Vietnam’s medical camps are necessary to improve patient health conditions.

## Data Availability Statement

The raw data supporting the conclusions of this article will be made available by the authors, without undue reservation.

## Ethics Statement

The Ethics Committee approved all procedures of Traditional Medicine Hospital–Ministry of Public Security, Hanoi, Vietnam (No.664/QD-YHCT). The patients/participants provided their written informed consent to participate in this study.

## Author Contributions

KH, VT, and LV developed the study concept and provided critical revisions. KH, VT, LV, HT, MN, and HN performed the analysis. All authors contributed to draft the manuscript and to the study design and approved the final version of the manuscript for submission.

## Conflict of Interest

The authors declare that the research was conducted in the absence of any commercial or financial relationships that could be construed as a potential conflict of interest.

## Publisher’s Note

All claims expressed in this article are solely those of the authors and do not necessarily represent those of their affiliated organizations, or those of the publisher, the editors and the reviewers. Any product that may be evaluated in this article, or claim that may be made by its manufacturer, is not guaranteed or endorsed by the publisher.
